# Introduction of a novel mathematical model for the prediction of the preformed particle gel’s swelling in the presence of monovalent and divalent ions

**DOI:** 10.1038/s41598-024-53055-7

**Published:** 2024-02-08

**Authors:** Parviz Mehrabianfar, Mehdi Momeni, Farnam Razzaghi-Koolaee, Mohammad Eslahati, Pourya Malmir, Bahram Soltani Soulgani

**Affiliations:** 1 Department of Petroleum Engineering, Ahvaz Faculty of Petroleum, Petroleum University of Technology (PUT), Ahvaz, Iran; 2 Department of Research and Development, Farasakou Asaluyeh Company, Asaluyeh, Bushehr, Iran; 3 Department of Petroleum Engineering, Amirkabir University of Technology (AUT), Tehran, Iran; 4 Upstream Petroleum Industry Research Center, Research Institute of Petroleum Industry (RIPI), Tehran, Iran

**Keywords:** Environmental sciences, Hydrology, Chemical engineering

## Abstract

Excess water production is one of the challenges that can cause several operational and economic problems. In this work, a comprehensive study of the PPG swelling in the presence of monovalent and divalent ions was conducted. Then, a comprehensive and practical mathematical modified fractal grow (MFG) model that can calculate the amount of PPG swelling in different salinities overtime was introduced. The output of the model was compared with the experimental data and showed a matching of about 80%. The viscosity of the PPGs at various shear rates was studied and matched with the cross-viscosity model. To assess the thermal stability of the particle gels. The TGA result represented the 10% of weight loss up to the reservoir temperature. In the following, core flooding tests with different injection scenarios were conducted. The oil recovery for the water and water/PPG/water scenarios were 39.5% and 71.5%, respectively. Eventually, the relative permeability curves were plotted using the Corey approach, and the effect of the PPG injection on the relative permeability curves was shown. The PPG injection increased oil production and reduced the excess water production by reducing water mobility.

## Introduction

Production of water in oil and gas reservoirs is a crucial problem as the reservoir becomes mature. Veil et al. announced that 98 percent of substances that are produced might be water at the end of a petroleum reservoir's life^1^. Water production may cause early shut-in wells and wasteful production. In addition, water production can result in corrosion in facilities, scale deposition, and high cost for water and oil separation^2–4^. The more water is produced; the more costs are encountered. Globally, by the production of one barrel of oil nearly three barrels of water are produced. This condition is worst in the U.S., where 10 barrels of water are produced for the production of one barrel of oil. According to Bailey et al., the cost of removing and treating excess water could reach 40 billion US dollars globally. Therefore, controlling water production and water shut-off is a serious topic in the petroleum industry^5–7^.

One of the practical methods which is used on an industrial scale is chemical water shut-off treatment, and the most useful material due to its simple and inexpensive preparation is using of gel polymers. Gel treatment has been widely used in many countries around the world. Low cost and excellent efficiency are two advantages of this approach^8,9^.

However, gel treatment as a chemical treatment has its own limitations. Different factors including pH, temperature, particle size, and concentration must be considered when using this method. Since 1996, Preformed Particle Gels (PPGs) have been synthesized and successfully applied to control excess water production in some oilfields in China^10^. Previous research on PPGs has focused on swelling rate, swelling gel strength, and flow resistance^11^. Cost et al. (2000) defined three mechanisms of PPG movement in a micromodel^12^. Kim et al. (2003) studied the mechanical properties of PPG’s and the gel composition effect on swelling^13^. Kuzmichonok et al. (2007) used various gel systems to study the possibility of reducing excess water production and improving oil recovery^14^. Bai et al. (2007) studied the effects of gel compositions and reservoir environments on PPG’s gel strength and the swelling ratio^15^. They successfully synthesized a new type of PPG and tested the mechanism of PPG transport through porous media using etched-glass micromodels^16^. Elsharafi and Bai (2012) used the filtration test model and load pressure model to study the weak PPG effect on unswept, low-permeable zones during conformance control treatments^16^. Imqam et al. (2015) suggested methods for minimizing the penetration of PPG into the matrix^17^. Heidari et al. (2019) compared the oil recovery from fractured reservoirs, before and after using PPG in micromodel structures^18^. Hasankhani et al. (2019) investigated the water shut-off performance of asphaltene-augmented gel polymer in fractured oil reservoirs^19^. Mehrabianfar et al. (2020) performed a thorough, visual investigation into the performance of the PPG using a glass micromodel^20^. As mentioned, particle gels have advantages over polymer gels (in situ gels). Some of the advantages of the PPGs are mentioned in the following: (1) controlling the gelation time, (2) higher thermal and salinity resistance, (3) Swelling control ability. According to the mentioned properties, PPGs can be a suitable option for excess water production control, resulting in higher oil recovery. However, prediction and controlling the PPG swelling is an essential factor that should be noted before any injecting process^21–23^. Lack of information about swelling in different salinities at various times may cause the failure of the injection process and formation damage. Hence, a mathematical model that can anticipate the swelling under different salinity and temperature conditions at various times can be very beneficial. In this study, an attempt has been made to provide a simple and practical model so that this need can be answered in operational tasks with an appropriate error^24–26^.

Based on the previous sections, a thorough investigation of the mentioned problems during PPG injection has been conducted. The amount of the PPG swelling was first determined in different monovalent and multivalent salts. following the completion of the experimental measurements, it has been tried to provide a simple and practical mathematical model with an acceptable error. It is noteworthy that a suitable and user-friendly model should not have complicated parameters. Afterward, a comprehensive study on viscosity was conducted and the recorded data was matched with a suitable model. In order to perform an efficient PPG injection operation, the performance of the PPG should be evaluated under different thermal conditions. Eventually, the efficiency of the PPG treatment was assessed using core flooding tests. The results have also been analyzed by relative permeability curves.

## Material and procedure

The test procedures, materials, and apparatus that were utilized in this research are presented in the following sections:

### Crude oil and rock

**Figure 1:**
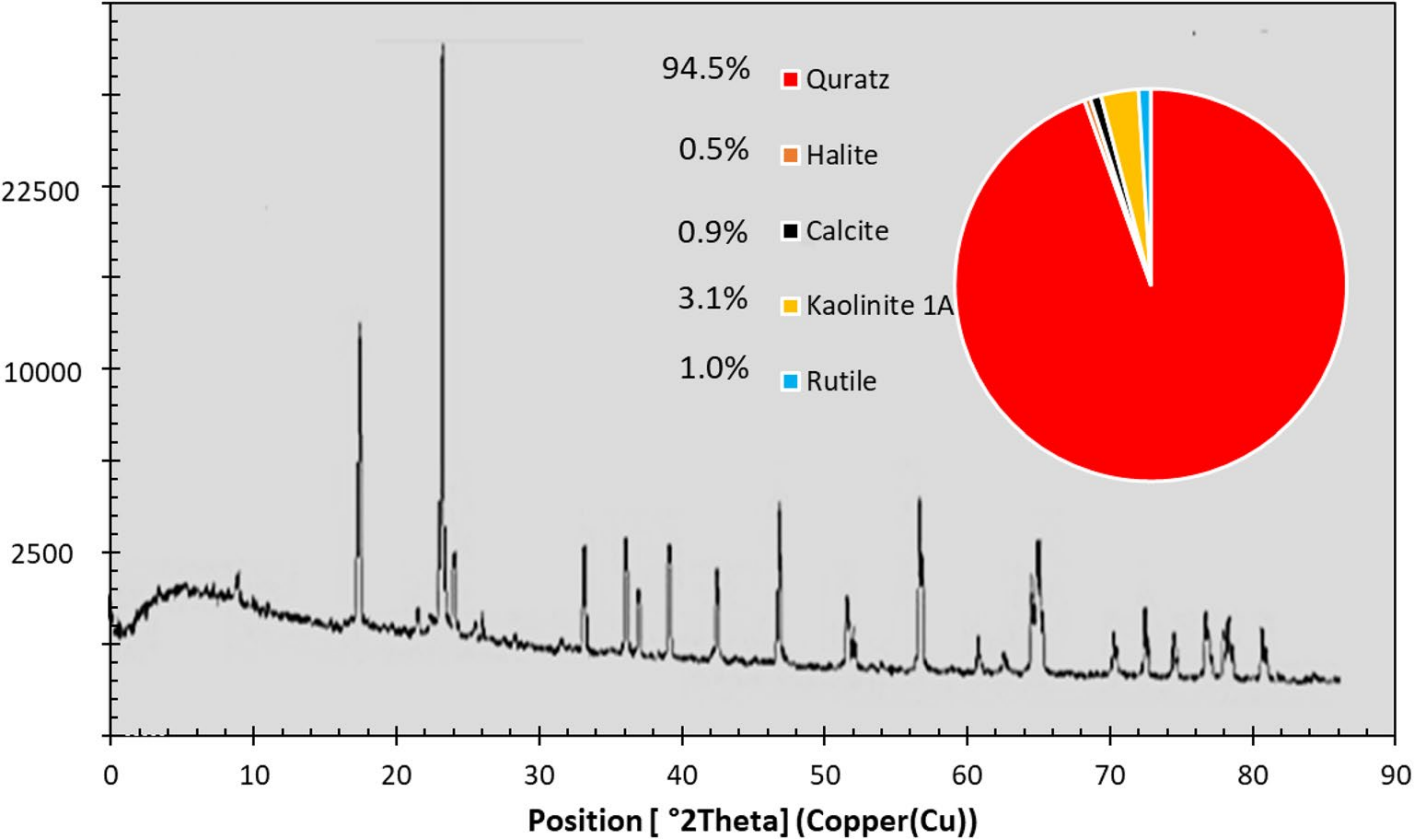
XRD analysis for rock sample.

The utilized crude oil was sampled from one of the oil reservoirs in the southwest of Iran. The percentage of crude oil components and the reservoir fluid properties are given in Table 1. The core plug was sampled from one of the sandstone oil reservoirs located southwest of Iran. X-ray Diffraction (XRD) analysis was performed on this rock sample and showed that it contains nearly 90% quartz, and the remaining constituent is mostly shale (Fig. 1).

**Table 1:** Cured oil properties used in this study.

Compound	C_3_	i-C_4_	n-C_4_	iC_5_	n-C_5_	C_6_	C_7_	C_8_	C_9_	C_10_	C_11_	C_12+_
Mole percent	0.28	0.32	2.38	1.98	2.01	2.56	8.80	6.55	11.86	9.76	10.17	33.33
Reservoir pressure = 4653 psia B_o_ = 1.4684 bbl/STB Saturation pressure = 2816 psia B_o_ = 1.5010 bbl/STB					Oil gravity of residual oil = 22.12°API Density of total gas evolved = 1.1259 g/l

### Core flood analysis

**Figure 2:**
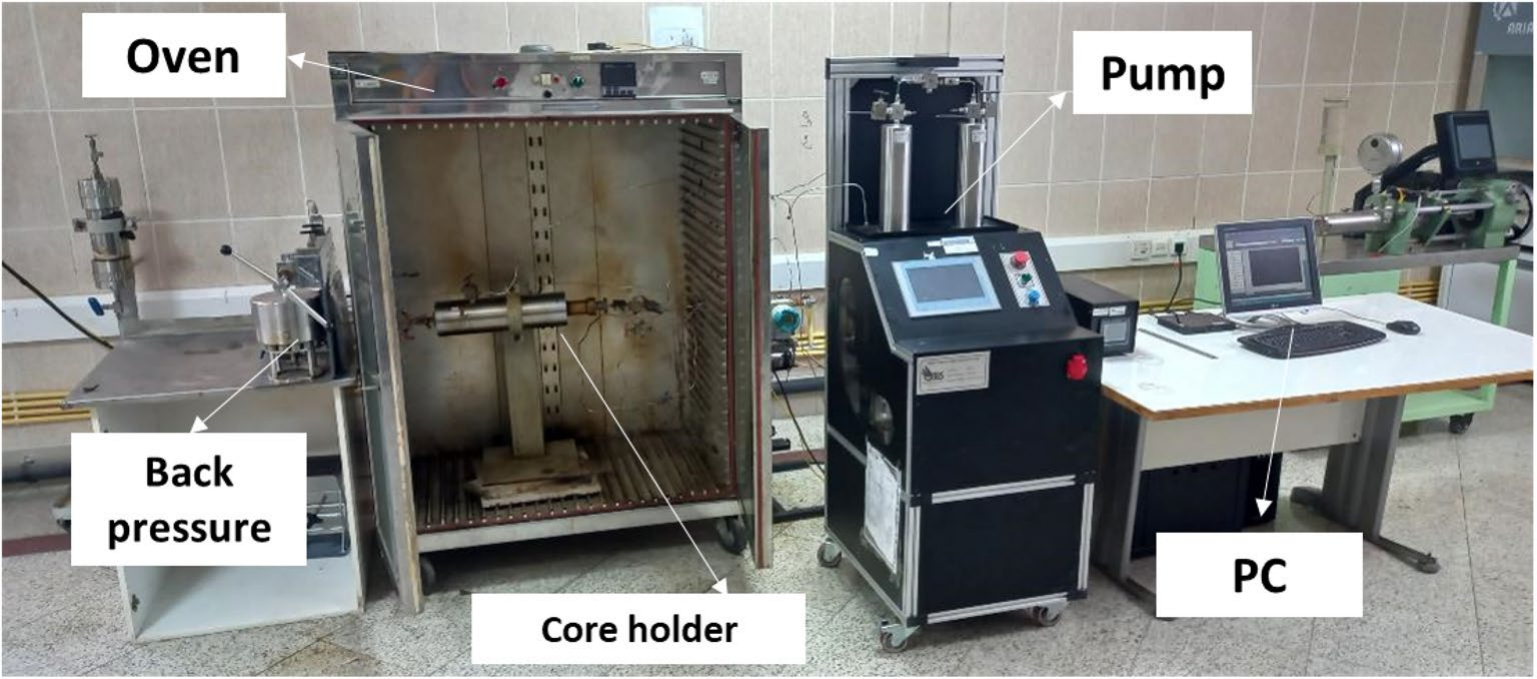
Schematic of core flood used in this study.

Flooding is one of the important experiments performed in this study to evaluate the performance of PPG under conditions close to the reservoir. In order to perform the test, the sandstone core was cleaned with toluene using a Soxhlet extractor apparatus for 24 h. The cleaned core sample was placed into the oven to get dried, and the weight of the dried core was recorded. Then the core sample was placed into the core holder and the formation water was injected into the sample to get completely saturated. By measuring the weight of the saturated core and the dry weight, the amount of the formation water in the pores of the core is calculated. The pore volume of the core sample is calculated by knowing the weight and density of the formation water^27–29^. The bulk volume of the core plug was also measured by recording the dimensions of the plug. The ratio of the pore volume to the bulk volume results in porosity. Subsequently, the saturated core plug was placed into the core holder and the crude oil was injected through the core until the water irreducible saturation (S_wi_) was achieved. Afterward, to create an oil-wet state in the core sample, it was aged into the crude oil for 2 weeks at a temperature of 50 °C^30,31^. After simulating the reservoir condition in the core plug, the core flooding experiment commenced at 75 °C. We have considered two scenarios for analyzing PPG behavior. In the first test, formation water was injected as the primary recovery process and the injection process was continued until no more oil was produced. For the second test, the formation water was injected to achieve the final primary recovery, then one pore volume of the PPG solution was injected through the core. The injection process was followed by the formation water. Figure 2 illustrates the schematic of the core flooding setup and the test procedure.

#### Relative permeability

In order to analyze the relative fluid flow, the relative permeability curve was plotted. Unsteady state method was applied to calculate the relative permeability. *Corey* approach was used to analyze relative permeability data. The methodology and relationships applied in this research are discussed below. Based on this method and the following formula (Eqs. 1 and 2), the relative permeability of the water and oil phase is determined^32–34^.

1 $$K_{rw} = \alpha \times \frac{{(S_{W} - S_{Wcrit} )}}{{(1 - S_{Wcrit} )}} - b \times (S_{W} - S_{Wcrit} ) \times \left[ {\frac{{(S_{wc} - S_{orw} )}}{{(1 - S_{Wcrit} - S_{orw} )}}} \right]^{2} + c \times \left[ {\frac{{(S_{wc} - S_{Wcrit} )}}{{(1 - S_{Wcrit} - S_{orw} )}}} \right]^{4}$$

2 $$K_{row} = d \times \frac{{(S_{o} - S_{orw} )}}{{(1 - S_{orw} )}} \times \left[ {\frac{{(S_{o} - S_{orw} )}}{{(1 - S_{wcon} - S_{orw} )}}} \right]^{2}$$

where $K_{rw}$ and $K_{row}$ are the relative permeabilities of water and oil phase, water and oil saturation are represented by $S_{W}$ and $S_{o}$, respectively, $S_{Wcrit}$ stands for critical water saturation, $S_{orw}$ represents the residual oil saturation post water flooding, and connate water saturation is shown by $S_{wcon}$. The coefficients a, b, c, and d change for various complexity of the crude oil, brine, and rock conditions.

### Swelling model procedures-modified fractal grow (MFG)

Gel swelling is one of the critical issues that should be considered during the injection. A mathematical model to predict the amount of swelling in various salinities and as a function of time is essential for modeling PPG in porous media. Hence, in this research, it has been tried to present a practical model for calculating the amount of swelling. Investigating the type and manner of PPG swelling and comparing it with the experimental data, represented a similarity between PPG swelling and asphaltene structure increment. Therefore, one of the approved models used in the field of asphaltene modeling was utilized to calculate the swelling potential of the PPG. The mathematical model is shown as below^35–37^:

3 $$R = R_{0} (1 + t/\tau_{D} )^{1/df}$$

where $\tau_{D}$ stands for diffusion time, t is flocculation time, $R_{0}$ represents the original particle size, $R$ is the mean radius of the asphaltene aggregates, and $df$ illustrates the fractal dimensionality.

There are two dominant mechanisms in this relationship according to the mentioned mathematical relationship. Diffusion-limited aggregation (DLA) and reaction-limited aggregation (RLA) are the two major mechanisms resulting from the irreversible aggregation of asphaltene within crude oil. The indicative property of DLA is the sticking of the particles together as a result of their contact. Hence, larger particle sizes are resulted according to flocculation time. However, the RLA mechanism represents that not each contact among particles causes aggregation and it is dependent on the number of particles in an aggregate. Therefore, much more collisions should occur for the aggregate formation and consequently, the aggregation rate is smaller than the DLA mechanism^38,39^.

**Figure 3:**
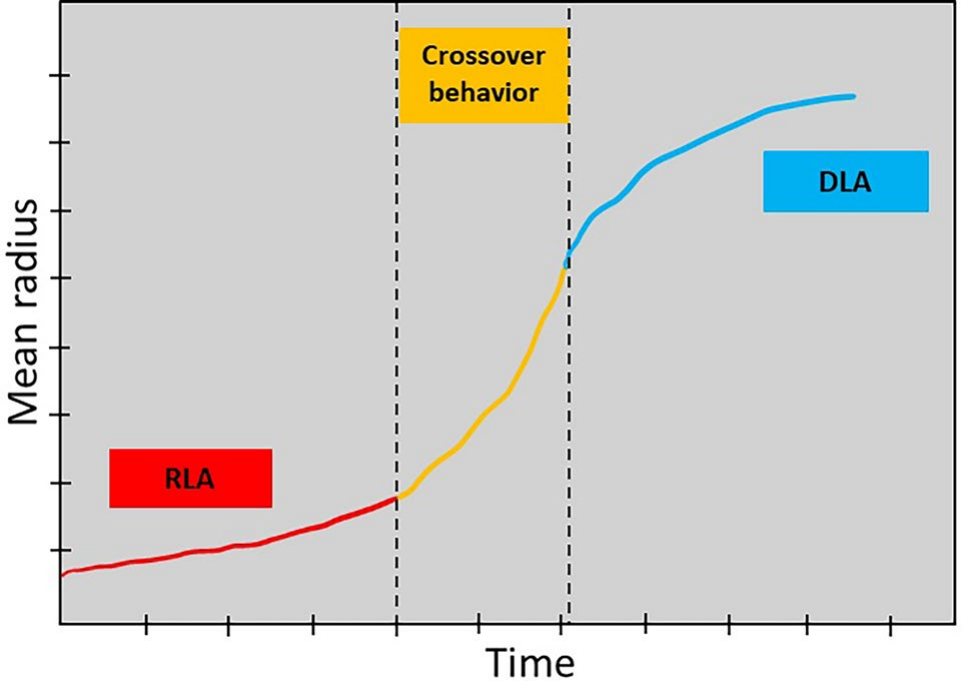
Asphaltene aggregation behavior.

According to Fig. 3 and checking the graphs and swelling data, it can be said that these data act like RLA and gel swelling can be modeled with this simple and practical mathematical relationship.

As aforementioned, based on the fractal model, time can be included in the relationship. To obtain a practical relationship for the gel swelling, this model was rewritten as follows:

4 $$SR = (Salinity)^{a} (1 + t/\tau_{D} )^{1/df}$$

It is clear from the mentioned relationship, that salinity and time are considered within the model which results in higher accuracy in gel swelling prediction [modified fractal grow (MFG)].

Considering that most natural phenomena have the same pattern, we checked multiple relationships and tried to use the simplest pattern of other phenomena. was the mostly matched relationship to the phenomenon. After detecting the suitable swelling pattern, a prominent mathematical relationship was presented. The novelty of our research was to introduce a simple model by identification of swelling patterns. The accuracy of the model was validated with different lab data from swelling tests and it ended in acceptable results.

## Result and discussions

### Swelling

Swelling is one of the basic parameters in determining the PPGs behavior and is very crucial in their performance. Observations showed that the performance of these gels in various salts was different. To investigate this behavior, we utilized three types of salt that are commonly found in the formation water^40–42^. The PPGs swelling in the presence of these salts and in different concentrations was evaluated by lab tests. Scanning electron microscopy (SEM) was applied to justify the impact of salts on the swelling of PPG. In addition, we generated mathematical modeling to predict the swelling behavior of the PPG. Finally, the PPG swelling results from lab measurements and mathematical modeling were compared.

#### Experimental

Investigating the amount of gel swelling in the presence of various salts with different concentrations.

##### ***Effect of Na***^+^***ion***

**Figure 4:**
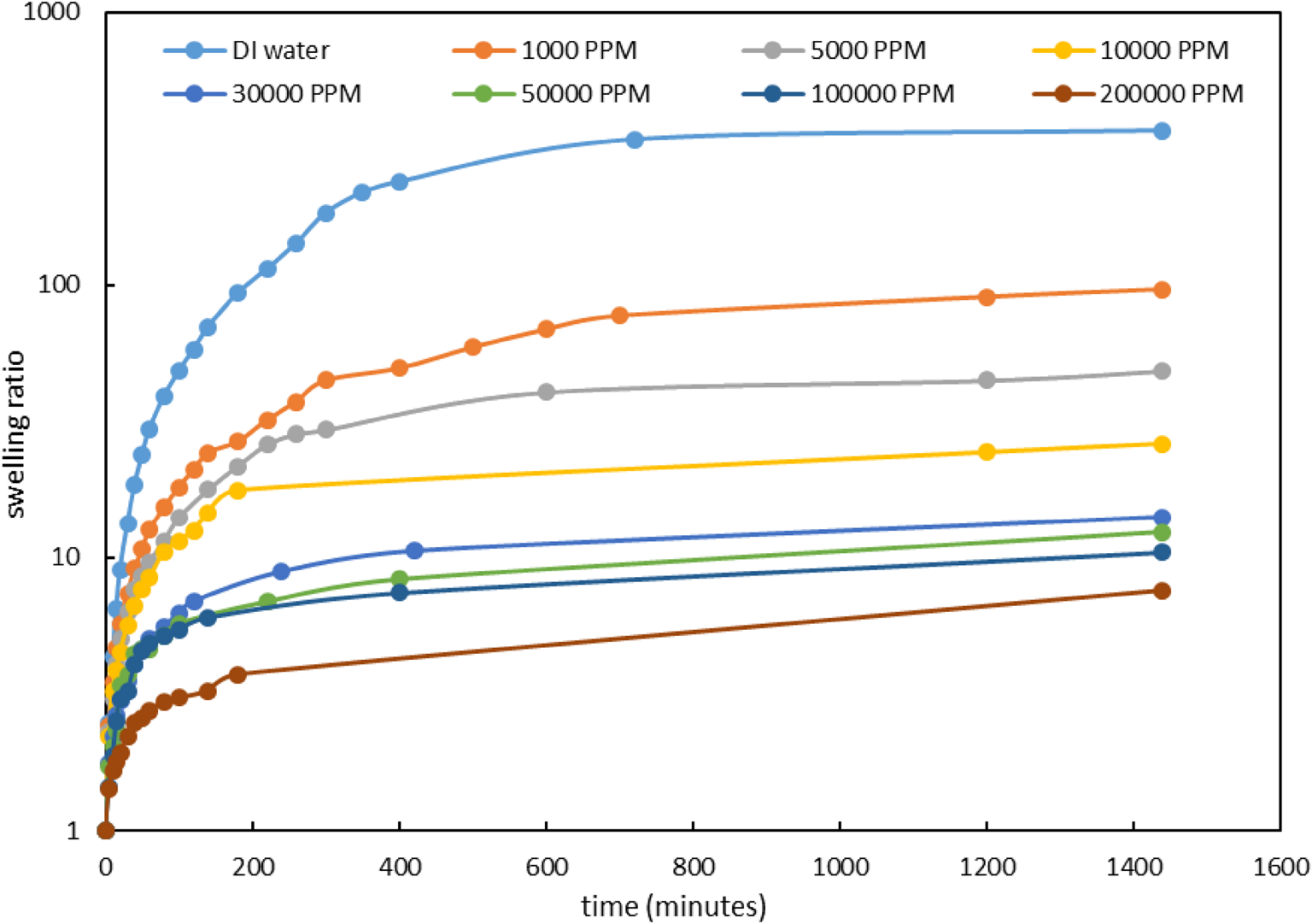
Swelling for PPG at different concentration of NaCl.

To peruse the effect of this ion, we prepared 8 different concentrations of NaCl. The salinities (1000, 5000, 10,000, 30,000, 50,000, 100,000, and 200,000 ppm) were set in such a way that a broad range of salinities is covered. The amounts of swelling shown in Fig. 4 were determined by measuring the weight of the gel at different times. The results demonstrated that the rate of swelling was high for the first 30 min, then slowed down, and finally, the water adsorption became constant after 3 h. The results clearly show that the gel swelling decreases with increasing water salinity. This reduction in swelling can be attributed to the physical and chemical adsorption of Na^+^ ions by gel. The adsorption of Na^+^ by gel reduces its tendency for water adsorption and consequently diminishes the swelling.

##### ***Effect of K***^+^***ion***

**Figure 5:**
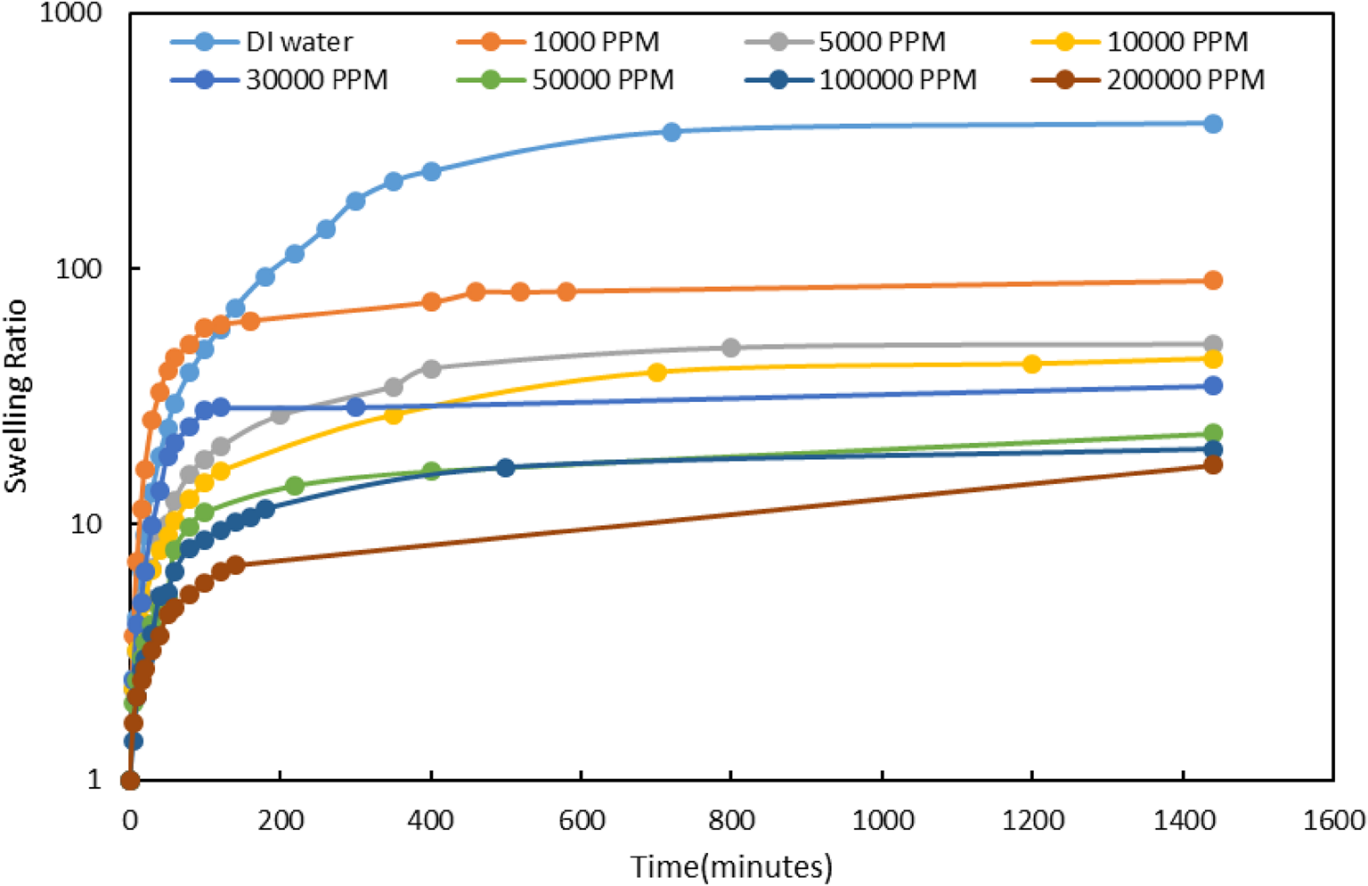
Swelling for PPG at different concentration of KCl.

In order to investigate the effect of monovalent ions on the gel swelling, the amount of swelling in the presence of KCl solution was also investigated. The salinities (1000, 5000, 10,000, 30,000, 50,000, 100,000, and 200,000 ppm) and amount of swelling is represented in Fig. 5, respectively. The results proved that swelling was reduced as a result of salinity increment, just like NaCl. Therefore, the swelling reduction mechanisms for both salts are identical. The maximum water adsorption occurs at first 20–40 min and the gel had about 10% less swelling in contact with potassium ions compared to sodium ions. The presence of potassium in the gel structure reduced the chemical adsorption factor which affected the swelling.

##### ***Effect of Mg***^***2***+^***ion***

**Figure 6:**
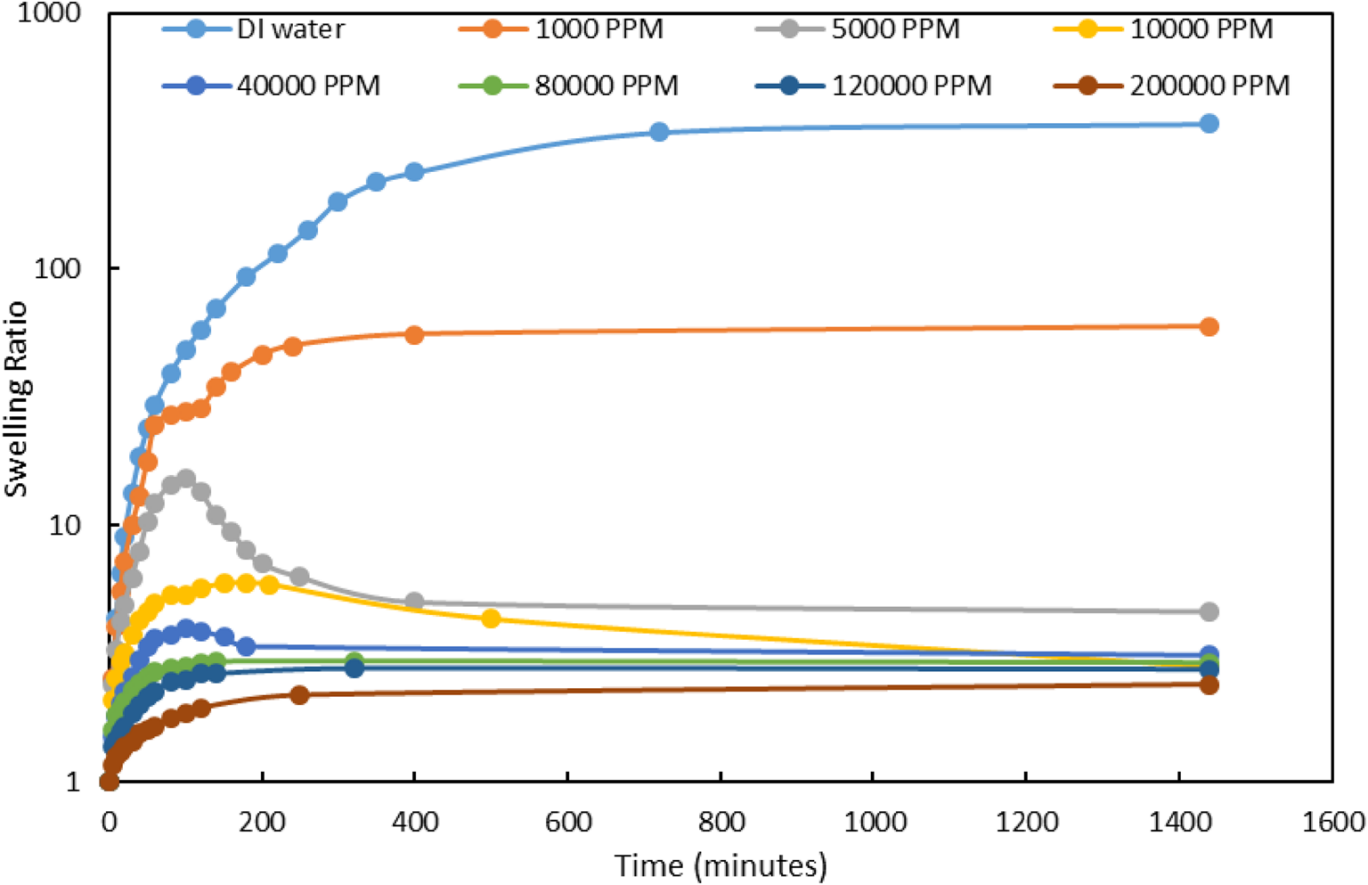
Swelling ratio for PPG in different concentration of MgCl_2_.

We utilized MgCl_2_ salt to compare the effects of monovalent and divalent ions on the gel swelling. The different MgCl_2_ salinities and the swelling result is shown in Fig. 6, respectively. The swelling was reduced after salinity increased like monovalent ions. According to Fig. 6 which represents the final gel swelling for three salts after one week, the gel swelling is the smallest value for MgCl_2_.This is because of Mg^2+^ ion hydration that adsorbs a small amount of water.

It is obvious that salinity will reduce the swelling capacity of the gel particles. The main reason that polymer networks can expand and create space for water absorption is anion-anion repulsion created by carboxylate and sulfonate groups in the polyacrylamide chain^43,44^. The presence of cations in the electrolyte solutions can reduce the anion-anion electrostatic repulsion, resulting in a lower swelling ratio^31,45^. By comparing the results, it can be demonstrated that MgCl_2_ can reduce the swelling ratio of PPG more than KCl. Because Mg^2+^ is a divalent cation, it can be more effective in reducing PPG absorption ability than K^+^ which is a monovalent cation.

According to the figures, the amount of swelling was measured within various salinity ranges. The main amount of swelling of various types of gels especially for PPGs occurs in the first hours of their contact with water. Based on the literature, they may swell up to 60% of their original volumes only in one hour after they were exposed to water. In this work, the pore size and swelling values were analyzed using some relations before PPG injection. Then the PPG can be injected through the medium at optimum salinity to avoid primary swelling problems. Consequently, the desired extent of swelling can be acquired by adjusting (lowering) the salinity of the injecting water.

##### SEM analysis

**Figure 7:**
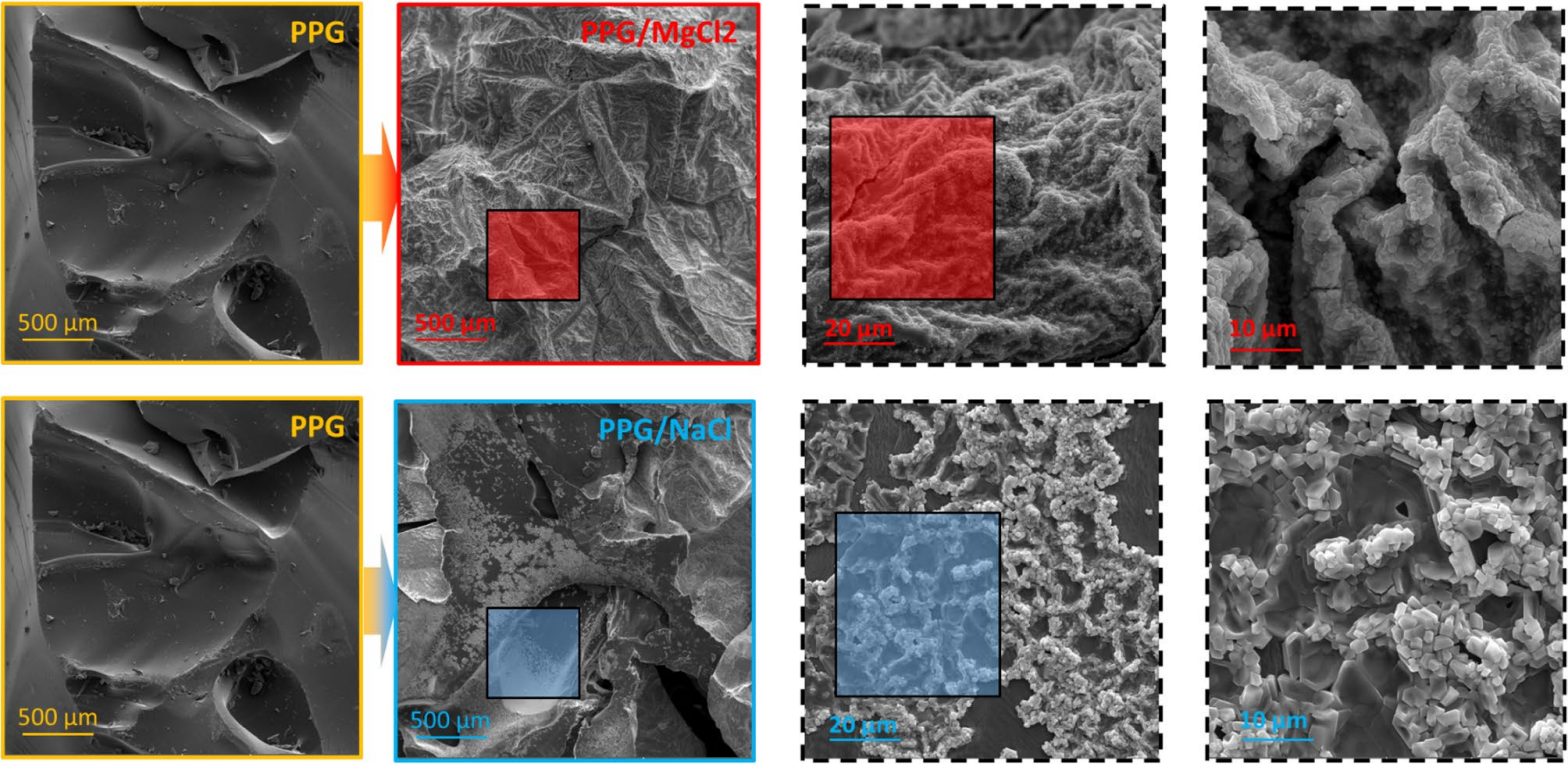
SEM analysis for PPG/NaCl and PPG/MgCl_2_ solution in different magnitude.

In this section, scanning electron microscopy (SEM) analysis was performed on the PPG in the presence and absence of salt solutions. The PPGs were aged in both DW and salt solutions for 2 days, and the maximum water adsorption occurred. The PPG samples were placed in the oven to dry slowly. Then the dried samples were utilized for SEM analysis. Figure 7 illustrate the surface morphology of PPG aged in NaCl and MgCl_2_ solutions, respectively. As it is obvious in these pictures, the PPG aged in the distilled water has a completely smooth surface. However, the salts were deposited on the surface of the PPG that was aged in saline solutions and demonstrating that the PPGs do not adsorb salts in their structure. Increasing the salinity results in higher salt deposition on PPGs and consequently lower PPG swelling (water adsorption).

#### Modeling

There have been different mathematical models for gel swelling in contact with water. However, there is no general model that covers the different salinity conditions and anticipates the gel performance. To generate an efficient model, we studied different models on swelling tests. It was found that the fractal growth model is most consistent with test results and accurately predicts swelling. Therefore, this modeling has been conducted on the test results of the gel swelling.

**Figure 8:**
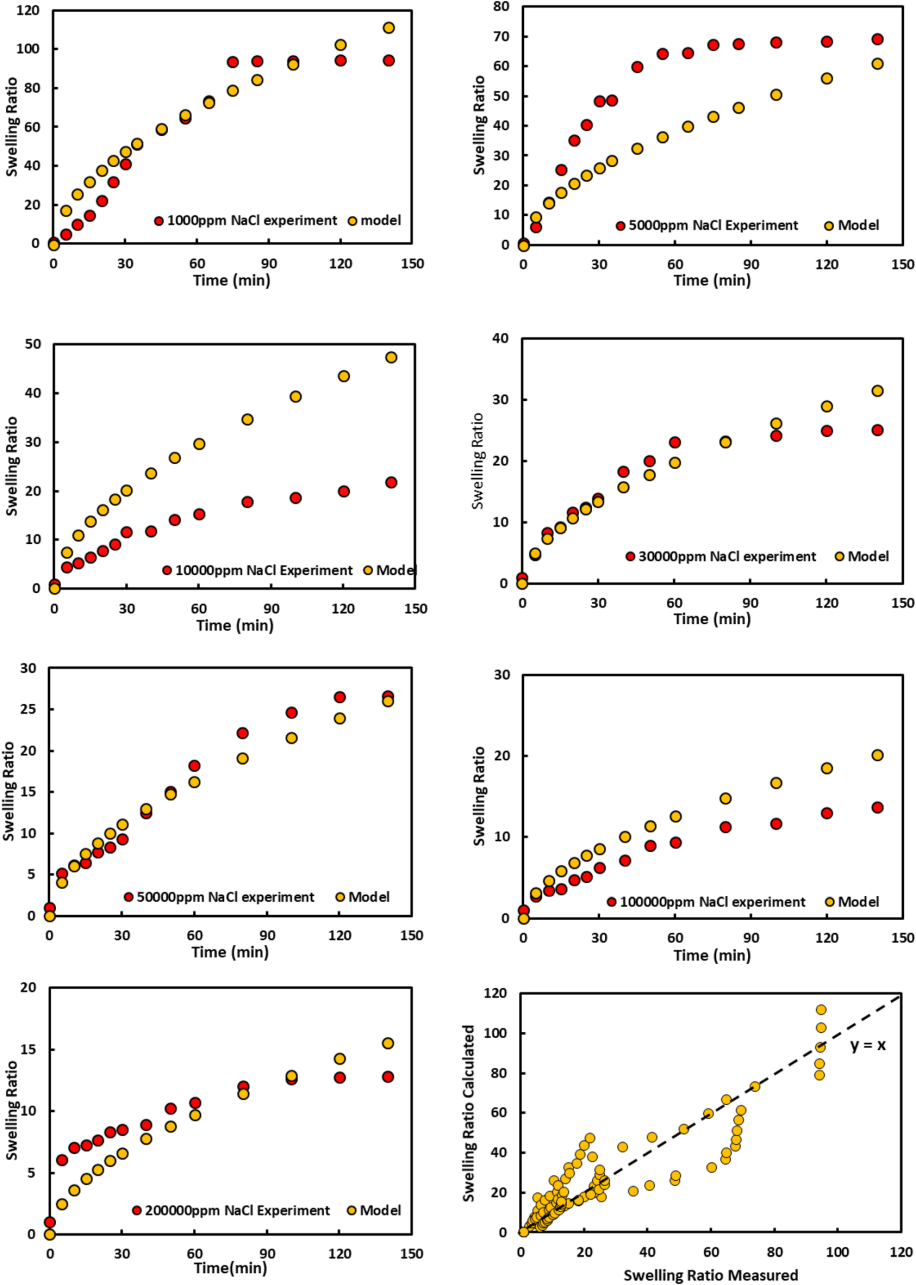
Experimental and modeling swelling ratio for PPG at different concentration of NaCl.

For instance, in Fig. 8, the real test data and swelling modeling results in presence of sodium ions are illustrated which indicates the efficient prediction of water adsorption and gel swelling by the model. By making a simple comparison and plotting the swelling ratio predicted by the model to swelling lab results, it was observed that the model has an excellent overlap with the real test results, and the regression coefficient of 0.8857 indicates this fact.

**Figure 9:**
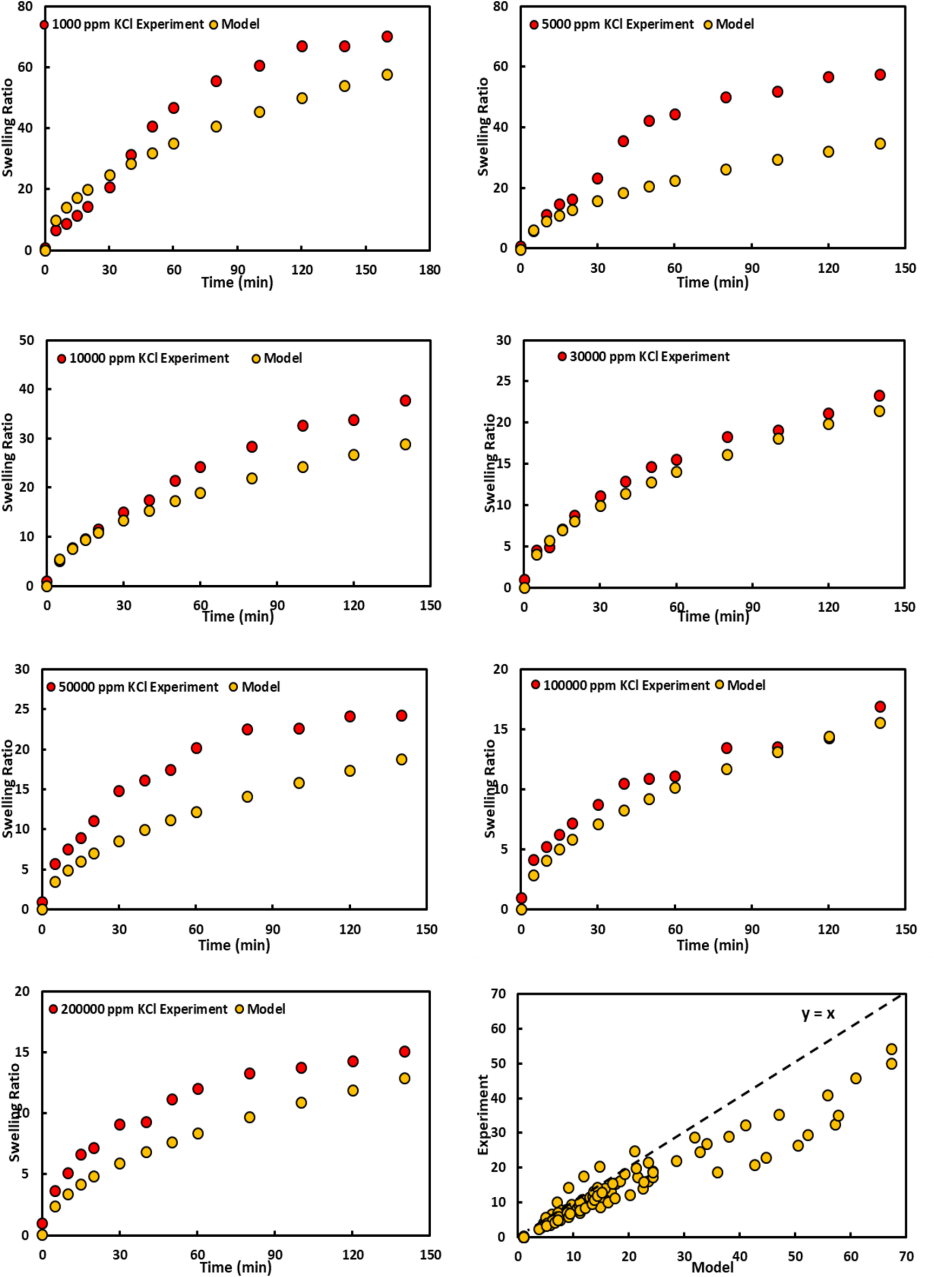
Experimental and modeling swelling ratio for PPG at different concentration of KCl.

**Figure 10:**
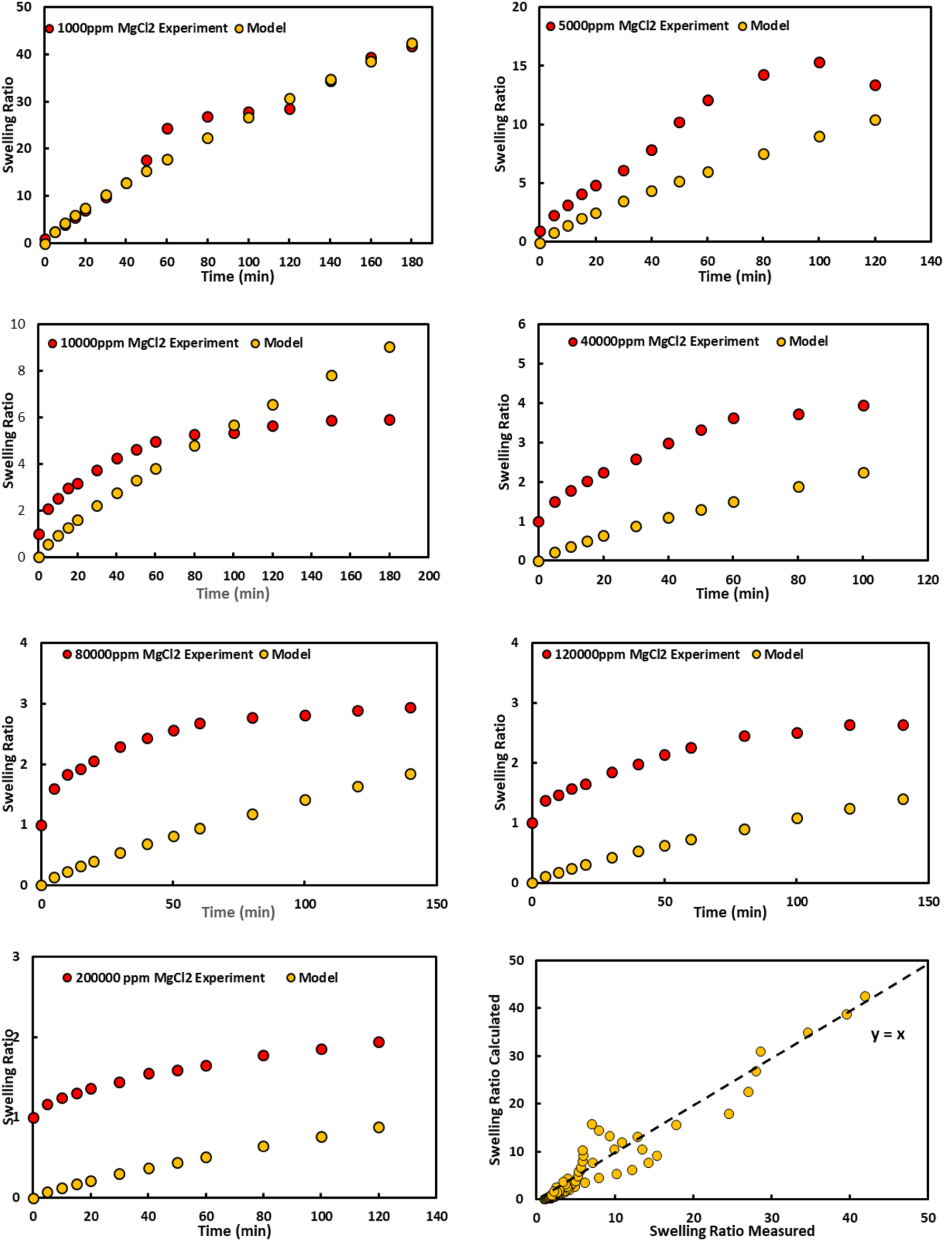
Experimental and modeling swelling ratio for PPG at different concentration of MgCl_2_.

Fractal modeling was also conducted on the results of KCl and MgCl_2_ salts. The ratio of the swelling predicted by the model to the real test is shown in Figs. 9 and 10, respectively. The regression coefficients for them are 0.83 and 0.89, respectively, which shows that the proposed model predicts the results of different salts as well as a wide range of salinity very well, and in fact, this model can be considered as the best model for predicting water absorption of the gels.

Generally, the results of gel swelling and their modeling revealed that increasing salinity, breaking the polymer structure, and reducing the physical water absorption reduce the gel swelling. Divalent ions have a considerable effect on the reduction of gel swelling. The swelling rate decreases sharply in the presence of divalent ions, but by looking at the results closely, it can be observed that even at very high salinity conditions, the gel absorbs water efficiently. Therefore, this performance gives an excellent option for water control operations. For this purpose, we studied the viscosity of the gel to justify its applicability as a water control agent (Table 2).

**Table 2:** Summary of model constant.

Salt	df	$\tau_{D}$	a	R^2^
NaCl	1.81	2.74E−04	−0.372210651	0.82
MgCl2	1.27	4.26E−03	−0.671220341	0.89
KCl	1.98	1.26E−03	−0.271220341	0.83

### PPG evaluation

In this part, different aspects of the PPGs that affect their performance are discussed thoroughly. Temperature stability, strength, and performance in the flooding test were carefully investigated for the PPG sample. It can be mentioned that all the aspects that are necessary for a successful PPG injection operation were examined.

#### Thermal stability

##### Swelling in different temperatures

The performance of the PPG is highly dependent on the temperature, and the stability of the swollen gel against temperature is a key parameter. Therefore, the stability of the PPG used at different temperatures was evaluated by measuring the amount of water adsorption and retention compared to the ambient temperature.

**Figure 11:**
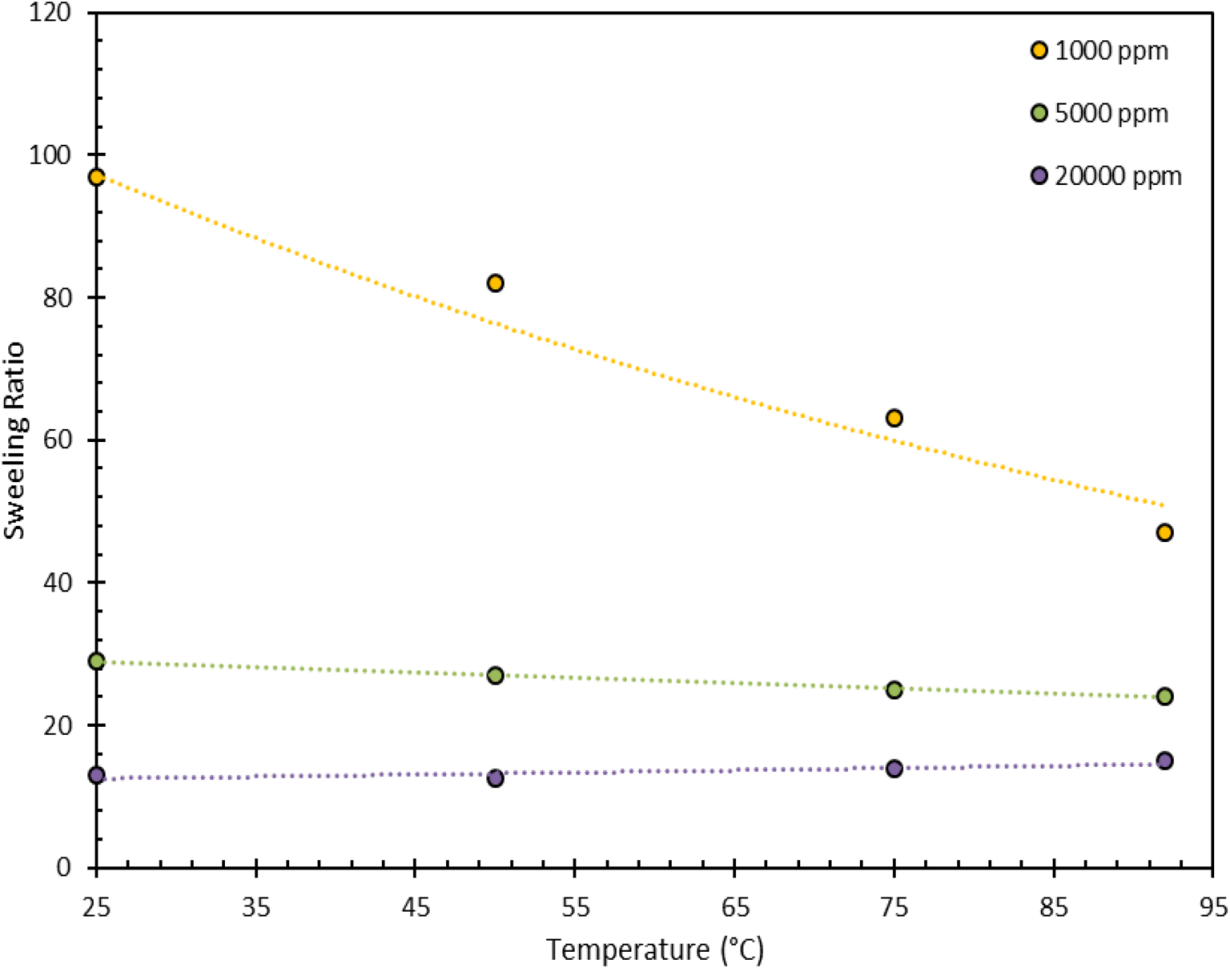
Swelling ratio for PPG at different concentration versus time.

For this purpose, the gel swelling at four concentrations was measured at four different temperatures. The water absorption was measured after 24 h of temperature exposure of the gel. Obviously, the swelling ratio has decreased with increasing temperature. The trend of the swelling ratio reduction is decreasing with salinity increase. (i.e., at higher salinities, the rate of swelling reduction with temperature increase is low). For instance, at a salinity of 220,000 ppm raising the temperature to 92 °C caused a reduction in swelling by less than 10%. This behavior indicates that high salinity, despite the many disadvantages and problems, has played a positive role and prevented the reduction of water adsorption by creating a barrier around the gel. Following the experiments, the gel samples were kept at a specific temperature for one month and the amount of water lost was calculated. We conducted this experiment to verify the stability of the gel under temperature over time. This assures that the gel used in the reservoir conditions does not lose its properties over a long period of time and retains its performance (Fig. 11).

##### TGA analysis

**Figure 12:**
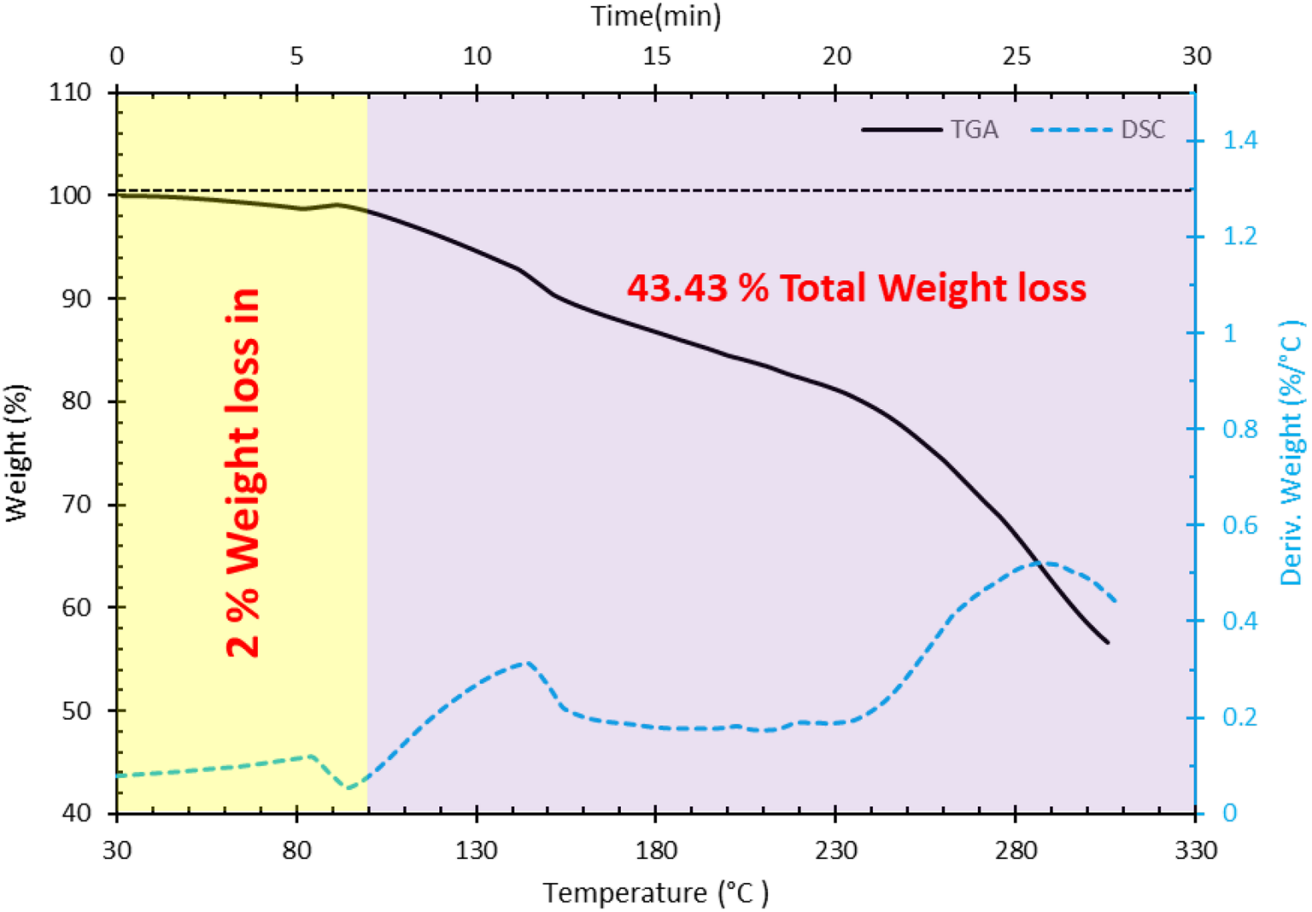
TGA analysis for determination of thermal stability.

Thermogravimetric (TGA) analysis was also utilized to evaluate the thermal stability of the PPG sample. SDT 650 Simultaneous Thermal Analyzer apparatus was used to perform this measurement. The temperature range for this experiment was between 25 and 300 °C and the temperature increase rate was 10 °C/min. An inert gas (Argon) was used to raise the temperature of the PPG sample^42,46,47^. As shown in Fig. 12, a total weight loss of about 43% was observed for this temperature range. According to the temperature range of oil reservoirs, which are mostly in the range of 90–95 °C, and based on Fig. 12, it can be mentioned that a maximum weight loss of 2% might be observed in the reservoir. Therefore, the PPG showed suitable stability and can be applied in real field operations.

#### Swelling in oil

**Figure 13:**
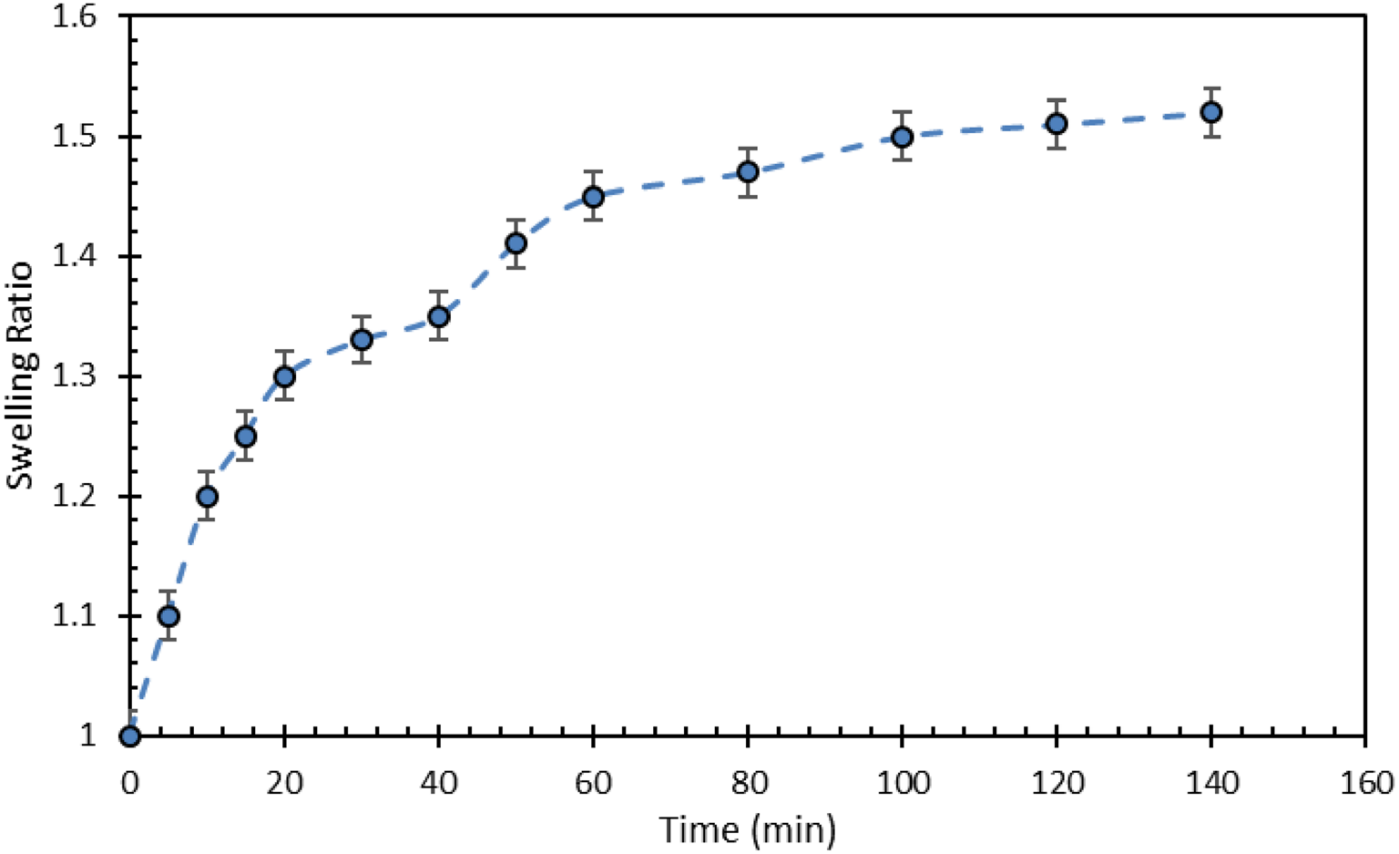
Swelling for PPG in crude oil.

PPGs are utilized to absorb water and prevent the production of excess water. So, if the gel also adsorbs hydrocarbons, there will be many problems, and PPG will not have the desired practical performance. In order to investigate the hydrocarbon adsorption potential of the PPG, a certain mass (1 gr) of the PPG was in contact with 50 cc of crude oil for a certain period of time, and the weight of the PPG was measured at different time intervals. Based on Fig. 13, crude oil could not enter the gel structure and cause swelling. The main mechanism of the gel swelling is chemical and hydrocarbon compounds (due to non-polarity) cannot enter the gel structure and expand it. Therefore, it can be applied to block water routes effectively. About 60–70% of the gel swelling process occurs in the first 1–2 h, and this causes problems in injecting it into the wells. As PPG does not adsorb hydrocarbons, the gel injectability problem can be solved by injecting it with gasoline in the well. After contact with the reservoir water, it starts to swell and closes the desired path. This method actually activates the intelligent water control mechanism by PPG.

#### Core flood analysis

Core flood tests through porous media were carried out to evaluate the performance of PPGs in recovery improvement. The first core flood test was performed as the reference case. The formation water was flooded through the core plug that was at the initial reservoir condition (water saturation = S_wi_). In this test, the injection was continued to ensure that the amount of produced and recovered oil reached its maximum value. Obviously, in the first 1 to 1.5 PV injection, the oil recovery has reached 90% of its final value. Finally, it was observed that the final oil recovery factor has reached approximately 40%. Also, the pressure drop diagram showed that there was a pressure drop at the beginning of the production and became constant after a specific time. Also, the relative permeability diagram for this test illustrated that the oil relative permeability decreased with a large slope at the beginning of the injection, and the water relative permeability diagram increased with a steep slope. It can be inferred from the relative permeability curve that the oil has a low ability to flow through porous media. By collecting and recording the volume of effluent fluids, the amount of associated water with the produced oil increased dramatically almost after 1 PV injection, relative permeability curve confirms this fact. The relative permeability curves elucidated that the relative permeability of oil is very low and it can be said that such injections are not possible on an operational scale.

**Figure 14:**
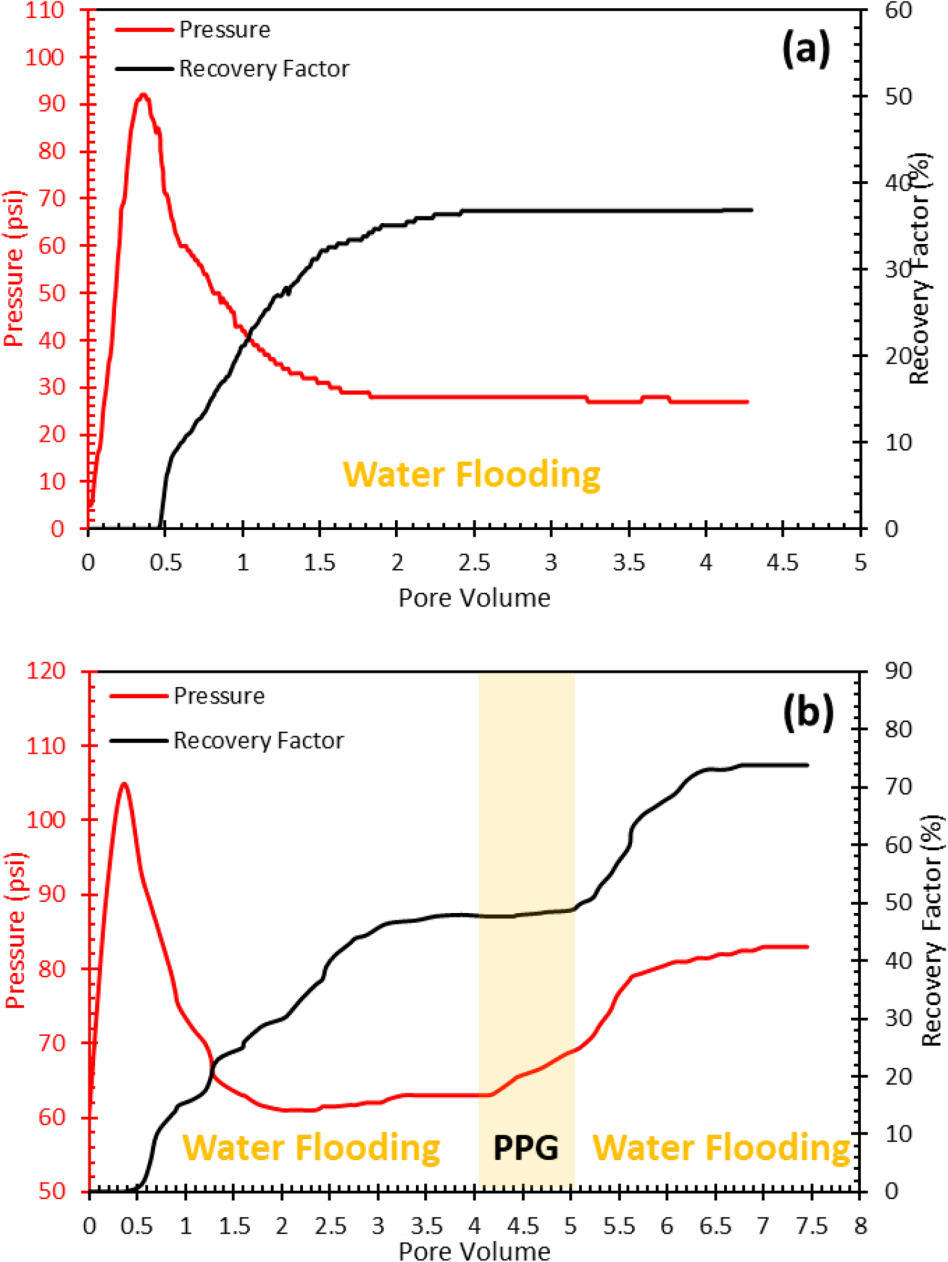
Recovery factor for water and water/PPG injection.

**Figure 15:**
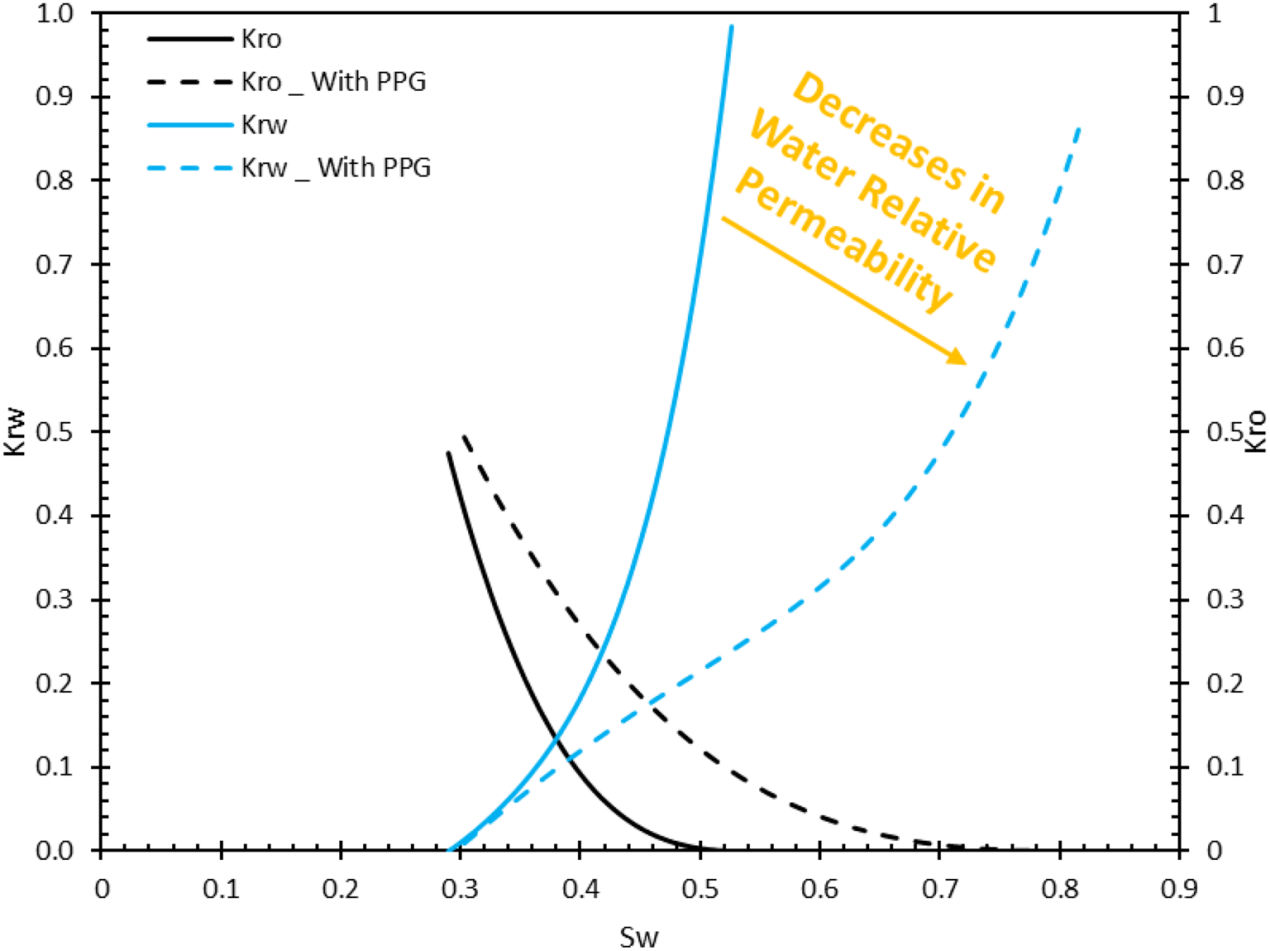
Relative permeability curve for core flood tests.

Another core flood test was performed with the same conditions, except 1 PV of PPG was injected after the oil recovery curve became constant. subsequently, the FW was injected after PPG injection. The differential pressure began to increase after PPG injection which means the pores and channels were blocked by PPG and excess water was adsorbed. The PPG injection causes two distinct phenomena. First, blocking the routes and channels by PPPs causes the injection front to be directed to the areas where the residual oil is left. The residual oil is stored in smaller pores and directing the injection front toward these areas causes an increase in injection pressure. Second, PPGs have a great potential to adsorb water due to their structural features. This property causes a great volume of water to be adsorbed and the relative permeability of water decreased. Consequently, oil moves easily and becomes produced. This feature also reduces the produced water to oil ratio and is more economical. Figure 14 represents the oil recovery versus PV of injected fluid and pressure demonstrates this fact. The relative permeability curves also confirm that the relative permeability of water has decreased and the relative permeability of oil has increased (Fig. 15). The PPG injection improved the oil recovery by 32%.

Therefore, it can be said that if the PPG injection plan is smartly devised (controlling the PPG swelling in various salinities at different times), the PPG performance will be higher and the produced water oil ratio will be diminished greatly (an efficient treatment).

## Conclusion

As mentioned previously, particle gels have acceptable thermal and salinity stability, gelation time control potential, and the ability to inject at specific concentrations. According to these features, a suitable injection pattern can result in a successful field operation for water control. In this study, a new mathematical model with appropriate accuracy and high efficiency was introduced, which represents matching of about 80%. Generally, to apply a particle gel injection scenario efficiently, the swelling potential of the gels should be determined at different salinity conditions with time. Lack of the proper mathematical model may result in blocking of the wellbore, formation damage, and also well abandonment. Here is the summary of the tests’ results:

- The amount of the particle gel swelling was higher in the presence of monovalent ions (K^2+^, Na^2+^) than divalent (Mg^2+^) ones. Because divalent cations have a greater potential for reducing PPG absorption. In addition, SEM analysis confirmed the difference in performance between monovalent and divalent cations.
- The proposed model (MFG) showed a suitable performance for different ions according to time and salinity. The R^2^ for the NaCl, KCl, and MgCl_2_ salt was 0.82, 0.83, and 0.89, respectively, which were acceptable values.
- Thermal stability evaluation illustrated a proper gel swelling at reservoir temperature conditions. The TGA test also showed a 10% weight loss of PPG up to reservoir temperature.
- The result of PPG swelling in the crude oil sample showed no hydrocarbon adsorption by PPG.
- Core flooding tests revealed an increase in oil recovery by up to 32%. The relative permeability curve showed that the gel injection diminished water mobility.

## Data Availability

The datasets used and/or analyzed during the current study available from the corresponding author on reasonable request.
